# Q fever in the Netherlands: public perceptions and behavioral responses in three different epidemiological regions: a follow-up study

**DOI:** 10.1186/1471-2458-14-263

**Published:** 2014-03-20

**Authors:** Marloes Bults, Desirée Beaujean, Clementine Wijkmans, Jan Hendrik Richardus, Hélène Voeten

**Affiliations:** 1 Municipal Public Health Service Rotterdam-Rijnmond, P.O. Box 70032, 3000 Rotterdam, LP, The Netherlands; 2 Department of Public Health, Erasmus MC, University Medical Center Rotterdam, P.O. Box 2040, 3000 Rotterdam, CA, The Netherlands; 3 National Institute of Public Health and the Environment, Centre for Infectious Disease Control, P.O. Box 1, 3720 Bilthoven, BA, The Netherlands; 4 Municipal Public Health Service “Hart voor Brabant”, P.O. Box 3166, 5203 DD ‘s-Hertogenbosch, The Netherlands

**Keywords:** Zoonotic infections, Q fever, Risk perception, Behavioral responses, General public, risk communication

## Abstract

**Background:**

Over the past years, Q fever has become a major public health problem in the Netherlands, with a peak of 2,357 human cases in 2009. In the first instance, Q fever was mainly a local problem of one province with a high density of large dairy goat farms, but in 2009 an alarming increase of Q fever cases was observed in adjacent provinces. The aim of this study was to identify trends over time and regional differences in public perceptions and behaviors, as well as predictors of preventive behavior regarding Q fever.

**Methods:**

One cross-sectional survey (2009) and two follow-up surveys (2010, 2012) were performed. Adults, aged ≥18 years, that participated in a representative internet panel were invited (survey 1, n = 1347; survey 2, n = 1249; survey 3, n = 1030).

**Results:**

Overall, public perceptions and behaviors regarding Q fever were consistent with the trends over time in the numbers of new human Q fever cases in different epidemiological regions and the amount of media attention focused on Q fever in the Netherlands. However, there were remarkably low levels of perceived vulnerability and perceived anxiety, particularly in the region of highest incidence, where three-quarters of the total cases occurred in 2009. Predictors of preventive behavior were being female, older aged, having Q fever themselves or someone in their household, more knowledge, and higher levels of perceived severity, anxiety and (self-) efficacy.

**Conclusions:**

During future outbreaks of (zoonotic) infectious diseases, it will be important to instil a realistic sense of vulnerability by providing the public with accurate information on the risk of becoming infected. This should be given in addition to information about the severity of the disease, the efficacy of measures, and instructions for minimising infection risk with appropriate, feasible preventative measures. Furthermore, public information should be adapted to regional circumstances.

## Background

Q fever is a zoonosis caused by the bacterium *Coxiella burnetii*. The primary reservoirs of the bacterium are farm animals, including goats, sheep, and cattle [1]. Acute Q fever typically presents as an influenza-like illness, but severe infections, like pneumonia and/or hepatitis, may also occur [2,3]. Approximately, 1-5% of all Q fever cases may progress to a chronic infection, which often leads to life-threatening endocarditis. Although Q fever is associated with substantial morbidity, mortality is uncommon (1-2% of cases) [1,4].

**Figure 1:**
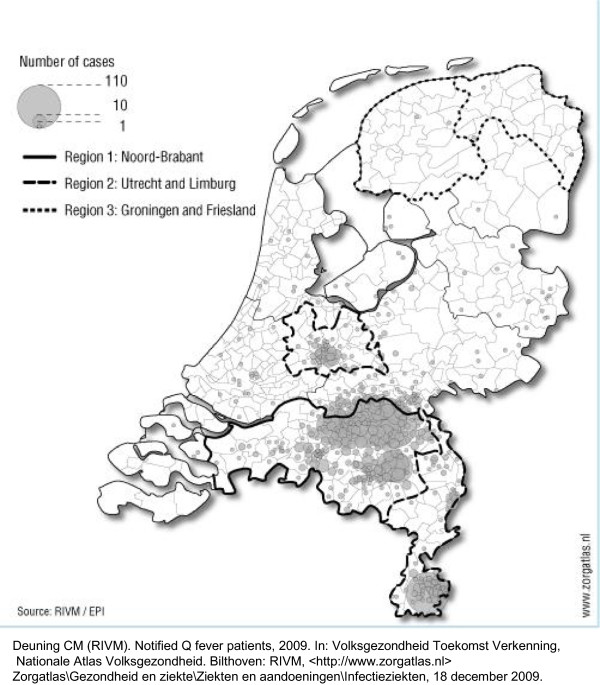
Notified patients with Q fever in 2009 (N = 2,357).

In the Netherlands, the first community outbreak of Q fever occurred in 2007, in the southern region of the Netherlands [5,6]. By the end of that year, 168 human Q fever cases were reported [7]. The second wave, in 2008, resulted in exactly 1,000 cases; in 2009, the number of cases reached a peak of 2,357 [7-9]. Research showed that the primary source of infection for humans was the wave of abortions on dairy goat farms, and that people that lived near these farms (within 5 km) were primarily affected [10]. As a result, the incidence of Q fever in the Netherlands differed between regions (Figure 1). In the first instance, Q fever was mainly a local problem of the Noord-Brabant province, which had a high density of large dairy goat farms. However, in 2009, an alarming increase in Q fever incidence was observed in adjacent provinces, including Utrecht and Limburg [8,11]. In 2009, the Dutch government decided to tackle the source by imposing various veterinary measures [8,12]. Furthermore, veterinarians, physicians, and the public were informed through targeted mailings, publications, and the news media. When a dairy goat or dairy sheep farm tested positive for *Coxiella burnetii*, all inhabitants living within a radius of 5 km of the farm received a letter to inform them of the presence of a Q fever-positive farm in their proximity. In 2011, patients with specific cardiovascular conditions and patients with aortic aneurysms or vascular prostheses that lived in high-risk areas were offered Q fever vaccinations [13]. These comprehensive measures have led to a significant decrease in the incidence of human cases (504 in 2010; 81 in 2011; 66 in 2012) [7].

Surveillance of public perceptions and behavioral responses during infectious disease outbreaks can provide useful information for designing health risk communications that achieve successful changes in public behavior [14,15]. Studies on public perceptions and behavioral responses have been conducted during outbreaks of other zoonotic infections, including severe acute respiratory syndrome (SARS) and avian influenza [16-23]. However, studies on public perceptions and behaviors during Q fever outbreaks have been limited, and they were mainly directed at specific risk groups [13,24,25]. The aim of the present study was to identify trends over time (2009, 2010, and 2012) and regional differences in public perceptions and behavioral responses, as well as predictors of preventive behavior, with regard to Q-fever.

## Methods

### Timing of the three surveys

The first survey took place from 13 August to 1 September, 2009. This followed a sharp increase in the incidence of human cases in spring 2009, primarily in the province of Noord-Brabant (as in 2007 and 2008), but it had also spread geographically to adjacent provinces (Figure 1). In late 2009 and early 2010, media attention markedly increased, and drastic veterinary measures were implemented. The second survey took place from 1 to 12 April 2010. This followed the period from January to May 2010, when 208 human cases were identified; this was lower than the number identified in the same period in 2009 [12]. The third survey took place from 2 to 17 April 2012, when the incidence had largely dropped off (66 cases in 2012) [7].

### Participants

The survey was conducted through an internet panel (the Flycatcher panel; http://www.flycatcher.eu), which retains national list of volunteers with the distribution of demographic variables (gender, age, region, and level of education) comparable to the general Dutch population. These volunteers can be invited to participate in online surveys. The Flycatcher panel meets high quality requirements and is ISO-certified. Panel members of three regions with different incidences of Q fever were invited to participate in this study. The regions included Noord-Brabant (with around 2464 000 inhabitants), which had the highest incidence of human Q fever, and Utrecht and Limburg (with around 2360 000 inhabitants), where Q fever had been more recently introduced. Two other provinces with low incidences of human Q fever, Groningen and Friesland (with around 1229 000 inhabitants), served as control regions (Figure 1). At three different time points, the participants completed an online survey. In the first survey, independent, random samples were selected for each geographical region; we invited a total of 2511 panel members (aged ≥18 years; about 800 per region). All respondents to the first survey were invited to participate in the second and third surveys. Sampled panel members were sent an email with an internet link. The surveys were available online for 5 to 10 days; during that time, panel members were required to respond. Upon completion of each survey, panel members received 1.50 Euro in credits, which could be exchanged for gift vouchers through the Flycatcher website.

This general, internet-based survey conducted with healthy volunteers from the general population did not require formal, medical ethical approval, according to Dutch law [26].

### Online questionnaire

The questionnaires were based on questionnaires used in similar studies on SARS, avian influenza, and Influenza A (H1N1) [18,21,22,27], with some modifications. The questions were based on an integrated model designed to explain health behavior. Constructs were used from the Protection Motivation Theory [28] and the Health Belief Model [29]; they included contact with the disease, perceived severity and vulnerability, feelings of anxiety, perceived efficacy of preventive measures, a persons’ ability (self-efficacy), intention to take measures, and actual preventive behavior. Participants were asked about eight (hypothetical) preventive measures against Q fever. Knowledge was examined with 7 true/false statements. The questionnaire concluded with items on the amount of information received on Q fever, attention paid to the information, and the reliability and sufficiency of governmental information. The questionnaires were similar across the three survey rounds (Additional file 1).

### Analysis

Data analysis was performed with SPSS for Windows, release 19.0. For all constructs with 3 or more items, Cronbach’s alpha was calculated (range 0.6 to 0.8). Therefore, for each construct, a summary score was calculated by summing the individual item scores and dividing by the number of items. For assessing knowledge, a summary score was created based on the number of correct answers (range 0–7). We computed the unadjusted bivariate correlations between the study variables using Cramérs V (for nominal vs. nominal/ordinal/interval variables) and Spearman’s Rho (for ordinal/interval vs. ordinal/interval variables). Paired t-tests (for comparing means) and McNemar tests (for comparing percentages) were used to analyse time trends between the baseline and first follow-up survey, and between the first and second follow-up surveys. Overall significant trends over time for the 3 regions are shown in the tables. Univariate logistic regressions were used to assess confounding factors. Comparisons of regional public perceptions and behavioral responses were analysed with ANOVAs and adjusted for the confounders; a p-value <0.05 was considered statistically significant. For each outcome variable, corrected regional means were calculated based on the results from the ANOVA model. Univariate and multivariate logistic regression analyses were performed to identify factors significantly associated with taking one or more preventive measures regarding Q fever. Prospective/follow-up studies are preferably used to identify a causal relationship between the predictors (measured at T1) and actual behavior (measured at T2) [30]. Therefore, we used data from the first survey in 2009 for the predictors in the regression analyses, whereas data from the follow-up survey in 2010 were used for the outcome variable (i.e. preventive behavior). For the multivariate regression analyses, all factors with a p-value <0.1 in the univariate analyses were entered in the multivariate model, and removed one-by-one (starting with the most insignificant one etc.) until only statistically significant predictors (p < 0.05) remained.

## Results

In August 2009, 2511 panel members were invited to participate. Of these, 64% (n = 1609) responded (baseline study). In the first follow-up survey, all 1609 respondents from the baseline study were invited, and 79% (n = 1263) responded. For the second follow-up survey, 1343 members of the 1609 respondents from the baseline study were invited, and 77% (n = 1032) responded. A total of 1347 respondents completed at least 1 follow-up survey and were included in the analyses. Of these, 932 completed both follow-up surveys. Significant differences were observed in the sex, age, education, employment, and marital status of participants in different regions; in the low incidence region a higher proportion of female, younger aged, higher educated, employed and single respondents participated compared to the high and intermediate incidence regions (Table 1). Univariate logistic regression analyses showed that sex, age, education, and employment status were statistically significant determinants (p < 0.05) in the majority of outcome variables, but not marital status.

**Table 1:** Demographic characteristics of respondents in each region (survey 1, August 2009)

**Characteristics**	**Region 1: high incidence region**^**a**^	**Region 2: medium incidence region**^**b**^	**Region 3: low incidence region**^**c**^	**Total**	***p*-Value**
	**(n = 459)**	**(n = 491)**	**(n = 397)**	**(n = 1347)**	
Sex					
Male	52%	53%	35%	47%	
Female	48%	47%	65%	53%	<0.001
Age					
18-30 years	14%	13%	26%	17%	
30-50 years	38%	43%	44%	42%	
Above 50 years	48%	44%	30%	41%	<0.001
Ethnicity^d^					
Native Dutch	91%	91%	93%	92%	
Immigrant	9%	9%	7%	9%	ns
Education^e^					
Low	31%	34%	16%	28%	
Intermediate	40%	35%	45%	40%	
High	30%	31%	39%	33%	<0.001
Employment status					
Employed	61%	62%	69%	64%	
Unemployed/Retired	39%	38%	31%	36%	0.04
Marital status					
Single	17%	19%	25%	20%	
Married/Cohabitating	80%	72%	69%	74%	
Divorced/Widowed	4%	9%	5%	6%	<0.001
Children < 18 years in household					
Yes	34%	37%	40%	37%	
No	66%	63%	60%	63%	ns

^a^Region 1 = Noord-Brabant – high incidence region; ^b^Region 2 = Utrecht & Limburg – intermediate incidence region; ^c^Region 3 = Groningen & Friesland – low incidence region;

^d^immigrant = born abroad or at least one parent born abroad; ^e^ low educational level (i.e. primary education, lower general/vocational education or less);

intermediate educational level (i.e. secondary general or vocational education); high educational level (i.e. higher professional education or university);

ns = not significant. Chi^2^ test was used to test demographic differences between regions.

### Correlations

Table 2 provides the unadjusted bivariate correlations between the study variables. Regarding the socio-demographic variables, for example, higher levels of perceived susceptibility, chance, anxiety and intention were observed among the lower educated (resp. p < 0.001; p < 0.001; p < 0.002; p < 0.001). However, low correlation values (<0.1 or between 0.1-0.3) were observed between the socio-demographic and cognitive variables. A couple of variables had moderate correlation values (between 0.3 and 0.5). Respondents with higher levels of perceived severity, susceptibility and chance also felt more anxious. A moderate correlation was also found between perceived susceptibility and chance, between perceived efficacy and self-efficacy/intention, and between perceived anxiety and preventive behaviour. A high correlation (0.78; p < 0.001) was found between self-efficacy and intention.

**Table 2:** **Bivariate correlations**^**a **^**for demographic and cognitive variables (survey 2010, n = 1249)**

	1	2	3	4	5	6	7	8	9	10	11a	11b	12	13	14	15	16
*Variable*^*b*^																	
9 Knowledge	.07	.09	.10	.16**	.09	.09	.09	.11*	1								
10 Perceived severity	.10	.16**	.08	.10	.17**	.11	.11	.09	.12**	1							
11a Perceived susceptibility	.04	.06	.05	.12**	.08	.07	.04	.14**	.11**	.23**	1						
11b Perceived chance	.09*	.07	.05	.14**	.07	.06	.07	.19**	.02	.11**	.42**	1					
12 Perceived anxiety	.10	.18**	.07	.14**	.17**	.13**	.06	.16**	.22**	.38**	.37**	.34**	1				
13 Perceived efficacy	.14	.16	.14	.18	.20*	.14	.14	.15	.14**	.14**	.09**	-.013	.19**	1			
14 Perceived self-efficacy	.19	.22**	.18	.18	.18	.16	.18	.15	.08**	.17**	.06*	-.007	.17**	.40**	1		
15 Intention	.14	.25**	.13	.20**	.21**	.17	.18	.19*	.09**	.26**	.13**	.06*	.29**	.43**	.78**	1	
16 Behaviour	.09	.13**	.07	.08	.11*	.06	.06	.25**	.13**	.20**	.21**	.14**	.38**	.20**	.20**	.28**	1

^a^Calculated using Cramérs V (for nominal vs. nominal/ordinal/interval variables) and Spearman’s Rho (for ordinal/interval vs. ordinal/interval variables);

^b^1 = gender (1: male; 2: female); 2 = age (1: 18–30 yrs; 2: 30–50 yrs; 3: <50 yrs); 3 = ethnicity (1: native dutch; 2: immigrant); 4 = education (1: low; 2: intermediate; 3: high); 5 = employment status (1: unemployed/retired; 2: employed); 6 = marital status (1: single; 2: married/cohabiting; 3: divorced/widowed); 7 = children in household (1: no children; 2: one or more children < 18 yrs); 8 = contact with disease (1: no; 2: had Q fever themselves/partner/child(ren); 9 = knowledge = 0–7 items correct; number 10–15 on header row relate to the corresponding number and variable presented vertically (1–5 point likert scale); 16 = behaviour (0–5 number of preventive measures taken).

*Correlation is significant at the 0.05 level (2-tailed); ** Correlation is significant at the 0.01 level (2-tailed).

### Trends over time (2009, 2010, 2012)

Public knowledge regarding Q fever increased significantly between 2009 and 2010, but slightly decreased between 2010 and 2012 (Table 3). Perceived severity increased over time from 2009 to 2010, and from 2010 to 2012. Perceived severity of the disease was rather high; the majority of respondents agreed that Q fever is a severe disease, that it is very harmful for their health, and that the consequence of getting Q fever in the coming year is (very) severe. The perceived personal susceptibility to Q fever remained stable over time, while the perceived chance of getting infected in the coming year decreased between 2010 and 2012. Perceived vulnerability generally was rather low, with less than 15% of respondents perceiving that they were susceptible. Perceived anxiety increased from 2009 to 2010, but decreased between 2010 and 2012 to the level of the first survey in 2009. Throughout this period, anxiety levels remained low (with less than 10% worrying about Q-fever).

**Table 3:** Trends over time in public perceptions and behaviors regarding Q fever in the Netherlands (2009, 2010, and 2012)

	**Survey 1**		**Survey 2**		**Survey 3**		**Trends over time**	
	**August 2009: baseline (n = 1347)**		**April 2010: first follow-up (n = 1249)**		**April 2012: second follow-up (n = 1030)**^**a**^		**Survey 1 versus 2**	**Survey 2 versus 3**
	**High score (%)**^**b**^	**Mean**	**High score (%)**^**b**^	**Mean**	**High score (%)**^**b**^	**Mean**	***p*-value**^**c**^	***p*-value**^**c**^
Knowlegde								
Summary score – Chronbach’s alpha 0.6	33	2.73	59	3.80	49	3.42	<0.001 (+)	<0.001 (−)
Perceived severity [scale 1–5]								
1. “Q fever is a severe disease”	57	3.53	73	3.79	78	3.89	<0.001 (+)	0.04 (+)
2. “Q fever is very harmful for my health”	53	3.45	63	3.65	67	3.73	<0.001 (+)	ns
3. Severity of getting Q fever coming year	57	3.67	70	3.94	77	4.08	<0.001 (+)	<0.001 (+)
Summary score – Chronbach’s alpha 0.7	--	3.55	--	3.79	--	3.90	<0.001 (+)	<0.001 (+)
Perceived vulnerability [scale 1–5]								
1. Perceived susceptibility for oneself	11	2.63	14	2.67	14	2.67	ns	ns
2. Perceived chance of getting infected coming year	2	2.22	3	2.20	1	2.03	ns	<0.001 (−)
Perceived anxiety [scale 1–5]								
1. Worried about Q fever	5	2.17	8	2.36	6	2.16	<0.001 (+)	<0.001 (−)
2. Fear for Q fever	3	2.11	5	2.23	4	2.12	<0.001 (+)	<0.001 (−)
3. Thinking of Q fever	1	1.74	1	1.98	1	1.66	<0.001 (+)	<0.001 (−)
Summary score – Chronbach’s alpha 0.8	--	2.01	--	2.19	--	1.98	<0.001 (+)	<0.001 (−)
Perceived efficacy [scale 1–5]								
1. Practice better hygiene	60	3.57	50	3.31	51	3.34	<0.001 (−)	ns
2. Avoid Q fever affected regions	64	3.64	75	3.92	80	4.10	<0.001 (+)	<0.001 (+)
3. Avoid contact with goats and sheep	81	4.13	85	4.25	84	4.28	<0.001 (+)	ns
4. Do not use raw dairy products	57	3.57	60	3.65	66	3.84	0.04 (+)	<0.001 (+)
5. Wear face mask	24	2.65	30	2.85	45	3.29	<0.001 (+)	<0.001 (+)
6. Move to place without Q fever	17	2.21	31	2.61	42	3.14	<0.001 (+)	<0.001 (+)
7. Seek medical consultation with onset of symptoms	59	3.57	55	3.46	51	3.42	<0.001 (−)	ns
8. Take antibiotics	34	3.01	32	2.93	36	3.11	0.047 (−)	<0.001 (+)
Summary score – Chronbach’s alpha 0.7	--	3.29	--	3.37	--	3.56	<0.001 (+)	<0.001 (+)
Perceived self-efficacy^d^ [scale 1–5]								
1. Practice better hygiene	88	4.32	84	4.22	82	4.21	<0.001 (−)	ns
2. Avoid Q fever affected regions	65	3.72	67	3.77	66	3.77	ns	ns
3. Avoid contact with goats and sheep	83	4.26	85	4.26	83	4.22	ns	ns
4. Do not use raw dairy products	71	3.94	71	3.95	70	3.92	ns	ns
5. Wear face mask	40	3.15	40	3.08	42	3.15	0.04 (−)	ns
6. Move to place without Q fever	9	1.86	12	1.99	13	2.10	0.001 (+)	0.005 (+)
7. Seek medical consultation with onset of symptoms	81	4.20	76	4.05	75	4.03	<0.001 (−)	ns
8. Take antibiotics	73	3.98	67	3.81	71	3.90	<0.001 (−)	ns
Summary score – Chronbach’s alpha 0.8	--	3.68	--	3.64	--	3.66	0.02 (−)	ns
Intention^d^ [scale 1–5]								
1. Practice better hygiene	86	4.33	81	4.17	80	4.15	<0.001 (−)	ns
2. Avoid Q fever affected regions	70	3.86	69	3.82	72	3.93	ns	0.01 (+)
3. Avoid contact with goats and sheep	84	4.29	83	4.24	82	4.22	0.03 (−)	ns
4. Do not use raw dairy products	70	3.97	70	3.93	71	3.95	ns	ns
5. Wear face mask	40	3.10	36	3.00	39	3.10	0.003 (−)	0.04 (+)
6. Move to place without Q fever	8	1.79	11	1.92	11	2.04	<0.001 (+)	0.003 (+)
7. Seek medical consultation with onset of symptoms	79	4.17	73	3.98	68	3.89	<0.001 (−)	<0.001 (−)
8. Take antibiotics	68	3.90	63	3.71	61	3.71	<0.001 (−)	ns
Summary score – Chronbach’s alpha 0.8	--	3.68	--	3.60	--	3.62	<0.001 (−)	ns

ns = not statistically significant; ^a^ 932 respondents participated in both follow-up surveys (331 of region 1; 350 of region 2; 251 of region 3); ^b^ percentage of respondents who scored 4–5 (except for knowledge: percentage of respondents who answered 4 or more out of 7 items correctly); ^c^ time trends based on p-values obtained using paired t-tests; ^d^ respondents were asked to imagine that governmental health institutes would recommend the preventive measure; ‘(+)’ indicates a significant increase over time p < 0.05; ‘(−)‘ indicates a significant decrease over time p < 0.05.

From 2009 to 2010 to 2012, an increase was observed in the overall perceived efficacy of measures for preventing Q fever. The measures with the highest perceived efficacies were avoiding contact with goats and sheep and avoiding Q fever-affected regions. Overall, perceived self-efficacy decreased between 2009 and 2010 and remained stable thereafter. Respondents felt most confident in practicing better hygiene and avoiding contact with goats and sheep.

Intentions to take preventive measures decreased between 2009 and 2010 and remained stable thereafter. Intentions were highest for practicing better hygiene and avoiding contact with goats and sheep. The percentage of respondents that had actually taken one or more measures for preventing Q fever increased significantly between 2009 and 2010 (from 22% to 30%), but decreased between 2010 and 2012 to the level of the first survey in 2009 (from 30% to 23%). The respondents most often reported avoiding contact with goats and sheep and practicing better hygiene.

Between 2009 and 2010, increases were observed in the amount of information respondents received on Q fever, the amount of attention paid to this information, and the perceived sufficiency of governmental information (data not shown). Between 2010 and 2012, decreases were observed in the amount of information respondents received and the attention paid to information on Q fever. The perceived reliability of governmental information on Q fever was stable over time (with almost half of respondents perceiving governmental information to be reliable).

### Regional differences

In 2009, 2010, and 2012, public knowledge regarding Q fever was highest in the high incidence region and lowest in the low incidence region (Table 4). Over time, there were no regional differences in the perceived severity of Q fever. Generally, perceived vulnerability was highest in the high incidence region and lowest in the low incidence region (although not always significant). In all three surveys, perceived anxiety was highest in the high incidence region and lowest in the low incidence region.

**Table 4:** Regional differences in public perceptions and behaviors regarding Q fever in the Netherlands (high, medium, and low incidence regions)

	**Survey 1 (August 2009) - baseline**				**Survey 2 (April 2010) - first follow-up**				**Survey 3 (April 2012)-second follow-up -**			
	**Region 1: high incidence (n = 459)**	**Region 2: medium incidence (n = 491)**	**Region 3: low incidence (n = 397)**	***p*-value**^**a**^	**Region 1: high incidence (n = 430)**	**Region 2: medium incidence (n = 456)**	**Region 3: low incidence (n = 363)**	***p*-value**^**a**^	**Region 1: high incidence (n = 354)**	**Region 2: medium incidence (n = 375)**	**Region 3: low incidence (n = 277)**	***p*-value**^**a**^
	**Mean**^**b**^	**Mean**^**b**^	**Mean**^**b**^		**Mean**^**b**^	**Mean**^**b**^	**Mean**^**b**^		**Mean**^**b**^	**Mean**^**b**^	**Mean**^**b**^	
Knowlegde												
Summary score – Chronbach’s alpha 0.6	2.99	2.73	2.45	<0.001	4.02	3.67	3.68	0.001	3.55	3.44	3.22	0.04
Perceived severity												
1. “Q fever is a severe disease”	3.50	3.58	3.49	ns	3.75	3.81	3.80	ns	3.90	3.90	3.86	ns
2. “Q fever is very harmful for my health”	3.44	3.48	3.42	ns	3.64	3.65	3.66	ns	3.69	3.76	3.75	ns
3. Severity of getting Q fever coming year	3.68	3.68	3.65	ns	4.02	3.95	3.84	0.03	4.11	4.12	4.02	ns
Summary score – Chronbach’s alpha 0.7	3.54	3.58	3.52	ns	3.80	3.81	3.77	ns	3.90	3.93	3.88	ns
Perceived vulnerability												
1. Perceived susceptibility for oneself	2.73	2.60	2.58	0.003	2.73	2.67	2.61	ns	2.72	2.72	2.54	0.003
2. Perceived chance of getting infected coming year	2.73	2.67	2.60	ns	2.29	2.19	2.11	0.02	2.12	2.10	1.87	<0.001
Perceived anxiety [scale 1–5]												
1. Worried about Q fever	2.25	2.18	2.10	0.02	2.47	2.31	2.30	0.004	2.25	2.18	2.06	0.02
2. Fear for Q fever	2.16	2.09	2.07	ns	2.29	2.22	2.18	ns	2.20	2.14	2.03	0.03
3. Thinking of Q fever	1.91	1.68	1.60	<0.001	2.12	1.94	1.86	<0.001	1.76	1.67	1.54	<0.001
Summary score – Chronbach’s alpha 0.8	2.11	1.98	1.92	<0.001	2.29	2.15	2.12	<0.001	2.07	2.00	1.88	0.001
Perceived efficacy [scale 1–5]												
1. Practice better hygiene	3.63	3.50	3.58	ns	3.31	3.28	3.36	ns	3.27	3.35	3.41	ns
2. Avoid Q fever affected regions	3.63	3.63	3.66	ns	3.90	3.91	3.96	ns	4.04	4.11	4.15	ns
3. Avoid contact with goats and sheep	4.22	4.17	3.98	<0.001	4.32	4.25	4.18	ns	4.29	4.28	4.27	ns
4. Do not use raw dairy products	3.61	3.49	3.64	ns	3.61	3.65	3.70	ns	3.81	3.87	3.86	ns
5. Wear face mask	2.68	2.57	2.73	ns	2.83	2.79	2.95	ns	3.22	3.27	3.39	ns
6. Move to place without Q fever	2.26	2.03	2.37	<0.001	2.69	2.50	2.66	ns	3.19	3.01	3.24	0.04
7. Seek medical consultation with onset of symptoms	3.62	3.58	3.50	ns	3.53	3.39	3.46	ns	3.46	3.42	3.37	ns
8. Take antibiotics	3.07	2.95	3.01	ns	2.97	2.87	2.96	ns	3.11	3.11	3.13	ns
Summary score – Chronbach’s alpha 0.7	3.34	3.24	3.31	0.03	3.39	3.34	3.40	ns	3.55	3.55	3.60	ns
Perceived self-efficacy^c^ [scale 1–5]												
1. Practice better hygiene	4.38	4.31	4.27	ns	4.18	4.26	4.19	ns	4.25	4.18	4.20	ns
2. Avoid Q fever affected regions	3.57	3.73	3.90	<0.001	3.55	3.78	4.02	<0.001	3.58	3.81	3.99	<0.001
3. Avoid contact with goats and sheep	4.35	4.22	4.21	0.04	4.28	4.25	4.25	ns	4.23	4.19	4.26	ns
4. Do not use raw dairy products	3.99	3.90	3.95	ns	3.94	3.90	4.03	ns	3.84	3.94	4.05	0.03
5. Wear face mask	3.13	3.07	3.27	ns	3.02	3.00	3.27	0.003	3.07	3.12	3.31	0.04
6. Move to place without Q fever	1.74	1.76	2.13	<0.001	1.90	1.88	2.23	<0.001	2.00	2.04	2.28	0.006
7. Seek medical consultation with onset of symptoms	4.24	4.20	4.16	ns	4.06	4.01	4.07	ns	4.03	4.03	4.03	ns
8. Take antibiotics	4.04	3.96	3.95	ns	3.83	3.80	3.81	ns	3.91	3.87	3.92	ns
Summary score – Chronbach’s alpha 0.8	3.68	3.64	3.73	ns	3.60	3.61	3.73	0.008	3.62	3.65	3.76	0.04
Intention^c^ [scale 1–5]												
1. Practice better hygiene	4.39	4.32	4.25	0.046	4.20	4.22	4.06	0.048	4.18	4.17	4.09	ns
2. Avoid Q fever affected regions	3.77	3.87	3.97	0.04	3.69	3.85	3.95	0.006	3.82	4.02	4.00	0.02
3. Avoid contact with goats and sheep	4.39	4.30	4.19	0.01	4.29	4.23	4.20	ns	4.25	4.25	4.18	ns
4. Do not use raw dairy products	4.06	3.91	3.96	ns	3.95	3.87	3.99	ns	3.94	4.00	3.94	ns
5. Wear face mask	3.14	3.03	3.14	ns	2.92	2.94	3.17	0.01	3.06	3.12	3.17	ns
6. Move to place without Q fever	1.73	1.70	1.98	<0.001	1.80	1.87	2.12	<0.001	1.96	1.98	2.20	0.02
7. Seek medical consultation with onset of symptoms	4.23	4.15	4.12	ns	4.01	3.99	3.95	ns	3.92	3.89	3.85	ns
8. Take antibiotics	3.95	3.88	3.87	ns	3.73	3.68	3.73	ns	3.82	3.68	3.67	ns
Summary score – Chronbach’s alpha 0.8	3.71	3.65	3.69	ns	3.57	3.58	3.64	ns	3.62	3.64	3.64	ns

ns = not statistically significant; ^a^p-value obtained using ANOVA with sex, age, education, and employment status as confounders; ^b^means are corrected for differences in sex, age, education, and employment status;

^c^respondents were asked to imagine that governmental health institutes would recommend the preventive measure.

Regional differences were observed in perceived efficacy of measures only in 2009, with highest scores in the high incidence region and lowest in the medium incidence region. No regional differences were observed in perceived self-efficacy in 2009, but in 2010 and 2012 it was highest in the low incidence region. Regarding intention to take measures, no regional differences were observed over the three surveys. In all three surveys, respondents in the high incidence region most often took measures to prevent Q fever.

Regional differences were also observed in the reported amount of information received (all three surveys), in the amount of attention paid to that information (all three surveys), and in the perceived sufficiency of information provided by the government (2009). All amounts were highest among respondents in the high incidence region (data not shown). There were no regional differences in the perceived reliability of governmental information on Q fever.

### Predictors of preventive behavior

Univariate and multivariate logistic regression analyses were performed to identify predictors significantly associated with taking one or more preventive measure regarding Q fever (Table 5). From the multivariate logistic regression analysis, predictors of preventive behavior were being female (OR 1.4; 95% CI 1.1-1.8), older aged (<50 yrs: OR 2.0; 95% CI 1.3-3.1), having Q fever themselves/someone in their household (OR 5.4; 95% CI 1.0-28.1); more knowledge (OR 1.6; 95% CI 1.2-2.1), and higher levels of perceived severity (OR 1.6; 95% CI 1.2-2.1), feelings of anxiety (OR 2.3; 95% CI 1.7-3.1), efficacy (OR 1.7; 95% CI 1.3-2.2), and self-efficacy (OR 1.4; 95% CI 1.1-1.9).

**Table 5:** Predictors of preventive behavior regarding Q fever

	**% of respondents that took one or more preventive measures**	**Oddsratio (95%-CI)**^**§**^	
		**Univariate**	**Multivariate**
Sex			
Male	26.7	1.0	1.0
Female	32.6	1.3 (1.0-1.7)	1.4 (1.1-1.8)
Age			
18-30 yrs	17.9	1.0	1.0
30-50 yrs	27.4	1.7 (1.2-2.6)	1.6 (1.1-2.5)
< 50 yrs	37.0	2.7 (1.8-4.0)	2.0 (1.3-3.1)
Contact with disease			
No	29.5	1.0	1.0
Yes^#^	75.0	7.2 (1.4-35.7)	5.4 (1.0-28.1)
Level of knowledge			
0-3 items corectly answered	25.8	1.0	1.0
4-7 items correctly answered	37.7	1.7 (1.4-2.2)	1.6 (1.2-2.1)
Perceived severity			
Low perceived severity	21.4	1.0	1.0
High perceived severity	36.9	2.1 (1.7-2.8)	1.6 (1.2-2.1)
Level of anxiety			
Low perceived anxiety	17.1	1.0	1.0
High perceived anxiety	39.1	3.1 (2.4-4.1)	2.3 (1.7-3.1)
Perceived efficacy of measures			
Low perceived efficacy	22.2	1.0	1.0
High perceived efficacy	37.3	2.1 (1.6-2.7)	1.7 (1.3-2.2)
Perceived self-efficacy			
Low perceived self-efficacy	22.4	1.0	1.0
High perceived self-efficacy	36.2	2.0 (1.5-2.5)	1.4 (1.1-1.9)

^§^95%-CI 95% confidence interval; ^#^had Q fever themselves or someone in their household.

The following determinant are not included in this table, because they were not significant in the multivariate model (although they were univariate a significant predictor of preventive behaviour); education, ethnicity, employment status, marital status, and intention.

The following determinants were univariate not a significant predictor of taking preventive measures regarding Q fever: having children <18 years in household and perceived vulnerability (2 items).

## Discussion

Between 2009 and 2010, we found increases in the public knowledge, perceived severity, anxiety, and perceived efficacy of measures related to Q fever in the Netherlands. In the same period, increases were also observed in actual behavior, the amount of information received, the attention paid to the information, and the perceived sufficiency of government-provided information. These increasing trends coincided with marked increases in media attention to the Q fever outbreak in the Netherlands and in the drastic veterinary measures that were implemented in late 2009 and early 2010. Other studies also described an association between media coverage/the amount of information people received and the levels of public knowledge/risk perception [22,31]. Apparently, in April 2010 (when the first follow-up survey took place), the public was not well-informed on the reduced number of human cases during the spring of 2010. Perhaps public risk perception and preventive behavior had not yet decreased at that time, due to the increase number of fatal cases reported (7 in 2009; 11 in 2010) and the recent implementation of veterinary measures. In 2011 and 2012, the number of new human Q fever cases decreased further, largely as a result of the implemented veterinary measures [12]. Furthermore, at that time, media attention had decreased regarding the Q fever outbreak in the Netherlands. This may have led to the decreases (between 2010 and 2012) in public knowledge, perceived anxiety, preventive behavior, amount of information received, and attention paid to the information on Q fever.

Respondents in the high incidence region exhibited the highest levels of public knowledge, perceived anxiety, preventive behavior, amount of information received, and attention paid to the information. This was most likely due to the facts that this region had a high density of large dairy goat farms, had the first community outbreak of Q fever, and had the most human Q fever cases. Also, the local media in that region focused more attention on the Q fever outbreak.

Predictors of preventive behavior regarding Q fever were being female, older aged, having Q fever themselves/someone in their household, higher levels of knowledge, perceived severity, feelings of anxiety, and (self-)efficacy. So, besides rational arguments (such as perceived severity and efficacy of measures), emotional aspect like anxiety play a role in decision-making concerning preventive behavior.

We found a strong correlation between self-efficacy and intention to take preventive measures against Q fever. This is very much in agreement with other studies, that describe that threatening information only leads to preventive behaviour if efficacy beliefs are also high [32,33].

The perceived vulnerability and perceived anxiety were rather low, even in the high incidence region, during the peak of the outbreak. Other studies describe this finding as an “optimistic bias”, which could have an adverse effect on risk perception and public compliance [27,34,35]. It is important for the public to have an appropriate level of perceived vulnerability, because those that perceive themselves at risk are more likely to comply with government-advised preventive measures [16,18,36].

If worn properly, face masks are an effective intervention strategy in controlling an outbreak [37]. Studies conducted in Asia during outbreaks of SARS and avian influenza reported rather high levels of face mask use among the general public [16,20]. However, we found low levels of perceived (self-)efficacy and intention to wear face mask. Possible explanations are the fact that wearing a face mask has many practical barriers and appears to be associated with negative feelings, like disease victimization, and stigmatization.

A clear strength of this study was that data collection took place during an actual outbreak situation, in contrast to other studies, which used scenarios based on hypothetical situations. Another strength was that this study consisted of three repeated survey rounds; this enabled the analysis of trends over time. Moreover, we followed-up individuals; thus, the differences between survey rounds represented real trends over time and were not due to differences between study populations [38]. Furthermore, we used an online questionnaire, which created less of a social desirability bias than personal telephone interviews.

This study also had some limitations. First, the surveys took place in different months of the year (August in 2009 and April in 2010 and 2012). Although cases of Q fever can occur at any time of the year, most cases reported the onset of illness during the spring and early summer months, with peaks in April and May [8,12]. Our first survey took place during the summer, when the number of new human Q fever cases decreased. The second and third surveys took place during the spring, when the number of Q fever cases had increased. Thus, survey timing may have had some influence on public perceptions and behaviors. Second, the Internet panel was representative for the Netherlands as a whole with regard to gender, age and education. Our study population comprised inhabitants of three regions. Therefore, the results may not be generalisable to the whole country. Furthermore, participants were not fully representative for their region. Although gender, age and education were included as confounders when analysing regional differences in risk perception and preventive behavior, other results can be slightly biased (for example the percentages in Table 3). Third, samples were drawn from an Internet panel which often include ‘heavy internet users’ who are more likely to perform information seeking behavior. This might have led to some bias in the perceived amount of information received. Last, the fact that it was a follow-up study may have influenced participating respondents; after the first survey, they might have become more aware of Q fever in the Netherlands, and therefore, they might have paid more attention to information on Q fever in the media.

Our study had several implications for health authorities. First, when levels of knowledge, public perceptions, and/or behavioral responses are generally low, providing the public with more information through the media is expected to increase these factors. During future outbreaks of (zoonotic) infectious diseases, it will be important to provide the public with accurate and up-to-date information on the risk of becoming infected to instil a realistic sense of vulnerability. This should be given in addition to information about the severity of the disease, information on the efficacy of measures, and instructions for minimising infection risk with appropriate, feasible measures. Second, health communicators should take the public’s perceptions into account when formulating messages about the prevention of zoonotic infections; these messages should be adapted to regional circumstances. Therefore, surveillance of public perceptions and behavioral responses during outbreaks of infectious diseases is important. Furthermore, involving the public in risk communication or the decision-making process regarding the implementation of public preventive measures could have added value, because the public can provide important information, particularly about the (practical) feasibility of specific preventive measures. This is consistent with a previous evaluation report of the Q fever outbreak in the Netherlands, which stated that “the public should be more involved in the dilemmas of the government” [39].

## Conclusions

Overall, the trends over time and the regional differences in public perceptions and behaviors regarding Q fever appeared to parallel the trends in the number of new human Q fever cases in the different epidemiological regions in 2009, 2010, and 2012, and the amount of media attention on Q fever in the Netherlands during those years. However, the low levels of perceived vulnerability and perceived anxiety were remarkable, particularly in the high incidence region, with three-quarters of the total cases in 2009. During future outbreaks of (zoonotic) infectious diseases, it is therefore important to provide the public accurate information on the risk of becoming infected to instil a realistic sense of vulnerability. Furthermore, information should be adapted to regional circumstances. New research could focus on searching for the most effective methods (e.g., personalising risk) for providing this information during future outbreaks of infectious diseases.

### Data sharing

data are available on request from MB m.bults@rotterdam.nl

## Competing interests

The authors declare that they have no competing interests.

## Authors’ contributions

All authors contributed to the study design. MB, HV, DB, and CW played a primary role in the data collection. Data analysis was performed by MB and HV. MB and HV wrote the first draft of the manuscript; DB, CW, and JHR critiqued the manuscript and contributed to further drafts. HV is the guarantor. All authors read and approved the final manuscript.

## Pre-publication history

The pre-publication history for this paper can be accessed here:

http://www.biomedcentral.com/1471-2458/14/263/prepub

## Supplementary Material

Additional file 1 Survey questions ‘Q fever in the Netherlands: public perceptions and behavioural responses in three different epidemiological regions: a follow-up study’. Click here for file
