# Supplementary material for: Q fever in the Netherlands: public perceptions and behavioral responses in three different epidemiological regions: a follow-up study
Source: BMC Public Health. 2014 Mar 20;14:263. doi: 10.1186/1471-2458-14-263 (PMC4108011; doi:10.1186/1471-2458-14-263)
Supplement: Additional file 1 — Survey questions ‘Q fever in the Netherlands: public perceptions and behavioural responses in three different epidemiological regions: a follow-up study’. [file 1471-2458-14-263-S1.doc]

**Additional file**

**Survey questions ‘Q fever in the Netherlands: public perceptions and behavioural responses in three different epidemiological regions: a follow-up study’.**

**We would first ask you to answer some questions about your personal circumstances.**

1. **What is your country of birth?**
   - The Netherlands
   - Dutch Antilles/Aruba
   - Belgium
   - Germany
   - Indonesia
   - Morocco
   - Surinam
   - Turkey
   - Other, namely.............................................................................
2. **What is your mother’s country of birth?**
   - The Netherlands
   - Dutch Antilles/Aruba
   - Belgium
   - Germany
   - Indonesia
   - Morocco
   - Surinam
   - Turkey
   - Other, namely.............................................................................
3. **What is your father’s country of birth?**
   - The Netherlands
   - Dutch Antilles/Aruba
   - Belgium
   - Germany
   - Indonesia
   - Morocco
   - Surinam
   - Turkey
   - Other, namely.............................................................................
4. **Are you employed at the moment?**
   - Yes
   - No
5. **What is your marital status?**
   - Single
   - Cohabitating
   - Married
   - Divorced
   - Widowed
6. **Do you have children (younger than 18 years) in your household?**
   - No
   - 1 child
   - 2 children
   - 3-4 children
   - 5 or more children
7. **Did you or someone in your household ever had Q fever?**
   - No
   - Yes, myself
   - Yes, my partner
   - Yes, my child/children

**This survey is about Q fever.**

1. **Below statements are formulated about Q fever. Please indicate whether the following statements are right or false?**

|  | Right | False | Don’t know |
| --- | --- | --- | --- |
| - Q fever is caused by a virus |  |  |  |
| - Goats and sheep are the most important cause of Q fever for humans |  |  |  |
| - In the Netherlands, a vaccine is available that protects humans   against Q fever |  |  |  |
| - Q fever can be transmitted by inhalation of contaminated dust |  |  |  |
| - Eating/drinking raw dairy product can cause Q fever |  |  |  |
| - Antibiotics can reduce the symptoms of humans with Q fever |  |  |  |
| - In the Netherlands, people died following Q fever |  |  |  |

1. **Q fever is a severe disease.**
   - Totally disagree
   - Mostly disagree
   - Don’t agree or disagree
   - Mostly agree
   - Totally agree
2. **Q fever is very harmful for my health.**
   - Totally disagree
   - Mostly disagree
   - Don’t agree or disagree
   - Mostly agree
   - Totally agree
3. **A number of medical conditions are mentioned below. For each condition, please indicate how awful it would be if you were to be diagnosed with this condition in the coming year?**

|  | Not severe at all | Not severe | Even | Severe | Very severe |
| --- | --- | --- | --- | --- | --- |
| Seasonal influenza |  |  |  |  |  |
| Diabetes |  |  |  |  |  |
| Heart attack |  |  |  |  |  |
| Q fever |  |  |  |  |  |
| HIV or AIDS |  |  |  |  |  |
| Asthma |  |  |  |  |  |

1. **Do you think that, in general, you are susceptible to getting Q fever if you take no preventive measures?**
   - Not at all susceptible
   - Not really susceptible
   - Even
   - Quite susceptible
   - Very susceptible
2. How likely is it that you will be diagnosed with one of the following medical conditions in the coming year?

|  | Very unlikely | Unlikely | Even | Likely | Very likely |
| --- | --- | --- | --- | --- | --- |
| Seasonal influenza |  |  |  |  |  |
| Diabetes |  |  |  |  |  |
| Heart attack |  |  |  |  |  |
| Q fever |  |  |  |  |  |
| HIV or AIDS |  |  |  |  |  |
| Asthma |  |  |  |  |  |

1. **Are you worried about Q fever?**
   - Not at all worried
   - Not worried
   - A bit worried
   - Worried
   - Very worried
2. **Are you scared for Q fever?**
   - Not at all scared
   - Not scared
   - A bit scared
   - Scared
   - Very scared
3. **How often do you think about the Q fever?**
   - Not at all
   - Rarely
   - Sometimes
   - Often
   - Very often
4. **A number of preventive measures are mentioned below. For each measure, please indicate if you think it will prevent you from getting Q fever.**

|  | Certainly not | Probably not | Even | Probably | Certainly |
| --- | --- | --- | --- | --- | --- |
| Practice better hygiene (i.e. washing hands more often) |  |  |  |  |  |
| Avoid Q fever affected regions |  |  |  |  |  |
| Avoid contact with goats and sheep |  |  |  |  |  |
| Do not use raw dairy products |  |  |  |  |  |
| Wear face mask |  |  |  |  |  |
| Move to place without Q fever |  |  |  |  |  |
| Seek medical consultation with the onset of symptoms |  |  |  |  |  |
| Take antibiotics |  |  |  |  |  |

1. **Imagine that health authorities advice these measures. For each measure, please indicate if you think you would be able to take this measure.**

|  |  | Certainly not | Probably not | Even | Probably | Certainly |
| --- | --- | --- | --- | --- | --- | --- |
| Practice better hygiene (i.e. washing hands more often) | |  |  |  |  |  |
| Avoid Q fever affected regions | |  |  |  |  |  |
| Avoid contact with goats and sheep | |  |  |  |  |  |
| Do not use raw dairy products | |  |  |  |  |  |
| Wear face mask | |  |  |  |  |  |
| Move to place without Q fever | |  |  |  |  |  |
| Seek medical consultation with the onset of symptoms | |  |  |  |  |  |
| Take antibiotics | |  |  |  |  |  |

1. **Imagine that health authorities advice these measures. For each measure, please indicate if you intend to take this measure.**

|  |  | Certainly not | Probably not | Even | Probably | Certainly |
| --- | --- | --- | --- | --- | --- | --- |
| Practice better hygiene (i.e. washing hands more often) | |  |  |  |  |  |
| Avoid Q fever affected regions | |  |  |  |  |  |
| Avoid contact with goats and sheep | |  |  |  |  |  |
| Do not use raw dairy products | |  |  |  |  |  |
| Wear face mask | |  |  |  |  |  |
| Move to place without Q fever | |  |  |  |  |  |
| Seek medical consultation with the onset of symptoms | |  |  |  |  |  |
| Take antibiotics | |  |  |  |  |  |

1. **What have you done so far to prevent yourself from getting Q fever?**
   - Nothing
   - Practicing better hygiene (washing hands more frequently, using tissues when coughing or sneezing)
   - Avoiding contact with goats and sheep
   - Cancelled or postponed a visit to Q fever infected area
   - Did not use raw dairy products
   - Bought face mask
   - Sought medical consultation
   - Other, namely.............................................................................
2. **What is the amount of information you received about Q fever?**
   - No information
   - A little information
   - Some information
   - Much information
   - Very much information
3. **How much attention did you pay to the information about Q fever?**
   - Very little
   - Little
   - Even
   - Much
   - Very much
4. **Do you consider the information of the government on Q fever to be sufficient?**
   - Certainly not
   - Probably not
   - Even
   - Probably
   - Certainly
5. **Do you consider the information of the government on Q fever to be reliable?**
   - Certainly not
   - Probably not
   - Even
   - Probably
   - Certainly

**This is the end of the survey. Thank you for your cooperation. If you have any questions, don’t hesitate to contact us.**
